# The rise of big data: deep sequencing-driven computational methods are transforming the landscape of synthetic antibody design

**DOI:** 10.1186/s12929-024-01018-5

**Published:** 2024-03-16

**Authors:** Eugenio Gallo

**Affiliations:** 1 Department of Medicinal Chemistry, Avance Biologicals, 950 Dupont Street, Toronto, ON M6H 1Z2 Canada; 2 Department of Protein Engineering, RevivAb, Av. Ipiranga, 6681, Partenon, Porto Alegre, RS 90619-900 Brazil

**Keywords:** Antibody engineering, Antibody library design, Machine learning, Synthetic antibodies, Deep sequencing, Next-generation sequencing

## Abstract

Synthetic antibodies (Abs) represent a category of artificial proteins capable of closely emulating the functions of natural Abs. Their in vitro production eliminates the need for an immunological response, streamlining the process of Ab discovery, engineering, and development. These artificially engineered Abs offer novel approaches to antigen recognition, paratope site manipulation, and biochemical/biophysical enhancements. As a result, synthetic Abs are fundamentally reshaping conventional methods of Ab production. This mirrors the revolution observed in molecular biology and genomics as a result of deep sequencing, which allows for the swift and cost-effective sequencing of DNA and RNA molecules at scale. Within this framework, deep sequencing has enabled the exploration of whole genomes and transcriptomes, including particular gene segments of interest. Notably, the fusion of synthetic Ab discovery with advanced deep sequencing technologies is redefining the current approaches to Ab design and development. Such combination offers opportunity to exhaustively explore Ab repertoires, fast-tracking the Ab discovery process, and enhancing synthetic Ab engineering. Moreover, advanced computational algorithms have the capacity to effectively mine big data, helping to identify Ab sequence patterns/features hidden within deep sequencing Ab datasets. In this context, these methods can be utilized to predict novel sequence features thereby enabling the successful generation of de novo Ab molecules. Hence, the merging of synthetic Ab design, deep sequencing technologies, and advanced computational models heralds a new chapter in Ab discovery, broadening our comprehension of immunology and streamlining the advancement of biological therapeutics.

## Background

Natural antibodies (Abs) are the key components of an adaptive immune response; these are used to effectively target and neutralize immunogens. In the past decades, important breakthroughs have led to the engineering of synthetic Abs, also known as Ab mimetics or artificial Abs, where such synthetic proteins are designed to replicate the function of natural Abs [1]. Importantly, synthetic Abs are made fully in vitro, eliminating the requirement of an immunological response for their discovery and production [2]. These Abs offer several benefits over traditional ones (Table 1); these include reagent reproducibility, enhanced quality control, increased target affinity and specificity, improved protein stability and solubility, and customizable molecular features [3–5]. A further advantage is that their generation is less expensive to manufacture, with faster development times [6]. Overall their enhanced attributes permit for streamlined and adaptable processes, especially when considering the development of biological therapeutics. As such, the capacity to produce synthetic Abs has fundamentally transformed the current landscape of Ab generation.

**Table 1:** Major advantages of synthetic Abs over natural counterparts

Advantages	Description
Low production costs	Synthetic Abs can be manufactured in large quantities at a relatively low cost, unlike traditional monoclonal Abs, which are derived from animals and can be expensive to produce
Stability and production	Synthetic Abs can be engineered to exhibit greater stability promoting large-scale production when compared to natural Abs, which may be constrained by factors such as poor stability and host organism production
Reagent reproducibility	Synthetic Abs are highly reproducible, meaning each batch will have the same properties. This is important for research and diagnostic applications where precise measurements are required
Increased affinity and specificity	Synthetic Abs can be meticulously engineered to attain superior specificity and affinity for their target when compared to natural Abs. This attribute is crucial for various applications, including diagnostics and medicinal applications
Customizable antigen recognition site	The antigen recognition site of synthetic Abs can be precisely engineered to target any desired antigen. They can be customized to bind to a diverse array of targets, including those that pose challenges for natural Abs. Accordingly, synthetic Abs are well-suited for targeting specific molecules, including proteins, viruses, or bacteria
Humanization	Synthetic Abs can be engineered to be fully human, minimizing the risk of immune responses when used in therapeutic applications

In recent years, synthetic Abs have found a wide variety of biological uses; for instance, they have been highly disruptive in the medical field, particularly in molecular diagnostics and therapeutic applications [5, 7, 8]. Their advantages over natural Ab counterparts encompass the capability to efficiently produce large scale Ab molecules able to target specific epitope regions, including various antigen conformations. Moreover, synthetic Abs offer the ability to conduct sequential in vitro selections allowing to generate Ab molecules capable of binding shared epitopes across different antigens. Also, the rational design of synthetic Ab libraries offers opportunity to produce clonal variants with enhanced specificity and affinity. This contrasts with Abs obtained through immunization, which are constrained by the physiological mechanisms of B cell activation [1]. Moreover, in vitro synthetic Ab selections can be adapted to various selection conditions, such as pH levels, ions, and competing molecules. This flexibility aids in the production of potentially functional Abs tailored to specific environmental conditions/pressures. Furthermore, the synthetic Ab frameworks can be pre-defined, such feature allows for simplified downstream molecular re-cloning and directed evolution strategies [1]. Also, a pivotal characteristic of synthetic Ab platforms is their compatibility with automation and high-throughput screening platforms. As such, this property offers opportunity increases the feasibility to conduct deep sequencing studies and big data generation (Table 2). Overall, synthetic Abs have rapidly transformed the field of biotechnology and biological therapeutics, streamlining Ab development pipelines from lead identification to molecular optimizations.

**Table 2:** Global overview of major distinctions between natural versus synthetic Abs [1]

Properties	Natural Abs	Synthetic Abs
Experimentations	In vivo	In vitro
Associated technologies	Hybridoma	Molecular display (*e.g*. yeast, phage, ribosome)
Epitope binding	Broad	Selective
Output affinities	Limited	Expanded
Antigen conformation	Undefined	Defined
Sequential selections (for shared epitopes)	No	Yes
Defined selection conditions (e.g. pH, ions, competitors)	No	Yes
Molecular re-cloning (e.g. change format, add tag, dimerization, enzymatic fusion, autobiotinilation)	Difficult	Simple
Directed evolution (e.g. improve affinity, specificity, expression, stability)	Difficult	Simple
Ab framework	Limited	Broad
Amino acid positional frequencies and identities	No	Yes
Financial costs and resources	High	Low
Time for isolation and production	Long	Short
Integration into NGS platforms	Difficult	Easy
Amenable to automation	No	Yes
High-throughput	No	Yes
Overcome immunological tolerance	Difficult	Easy
Allows non-antibody scaffolds	No	Yes
Specificity for protein sequences and conformations	No	Yes
Target recognition (e.g. chemical modifications and small molecules)	Difficult	Simple

Another ground-breaking technology that has effectively disrupted biological research is deep sequencing, also known as next-generation sequencing (NGS) or high-throughput sequencing. This approach employs advanced molecular techniques that conduct DNA or RNA sequencing on a large scale with high efficiency. As a consequence, the fields of genomics and molecular biology research have undergone significant enhancements; for instance, deep sequencing has enabled the simultaneous sequencing of millions of DNA fragments or RNA molecules in a high-throughput manner. In this context, high-throughput sequencing offers a rapid, cost-effective, and efficient approach for sequencing complete genomes, exomes, and specific DNA segments [9–11]. As a consequence, this advancement allows for the swift and affordable execution of a wide range of molecular experiments associated with complex biological systems at an unprecedented level.

Furthermore, due to its rapid data acquisition, cost reduction, and ability to generate large datasets (Table 3), deep sequencing has found application in diverse scientific domains, including genomics, transcriptomics, epigenetics, metagenomics, and others. Its transformative impact is evident in biological research, where it has led to the effective discovery of novel gene variants, identification of disease-related biomarkers, and a comprehensive understanding of cancer-causing mutations [9, 12–15]. Recently, the integration of deep sequencing technologies into clinical diagnostics has revolutionized personalized medicine. Here, this integration has enabled the swift assessment of a patient's genetic profile, particularly genes relevant to disease susceptibility; furthermore, it has been effective in understanding cellular and molecular effects during treatment responses [16–18]. When taken together, deep sequencing is paving the way for important breakthroughs in both scientific research and medical applications [19].

**Table 3:** Several advantages associated with deep sequencing [10]

Advantage	Description
High-throughput	This technology can effectively sequence millions of DNA fragments in parallel; it generates massive amounts of sequence data during a sample run. As a consequence, it allows for the analysis of entire genomes, transcriptomes, and targeted regions of interest in a single experiment
Fast	A single NGS run can generate millions to billions of sequences in a matter of hours to days, whereas Sanger sequencing is much slower and labor-intensive
Cost-effective	It has the ability to sequence a large number of samples simultaneously, where the cost per base of sequence data is dramatically reduced when compared to traditional methods; this makes studies involving genomics and transcriptomics highly affordable
Accurate	Its current error rates are less than 1%

**Fig. 1:**
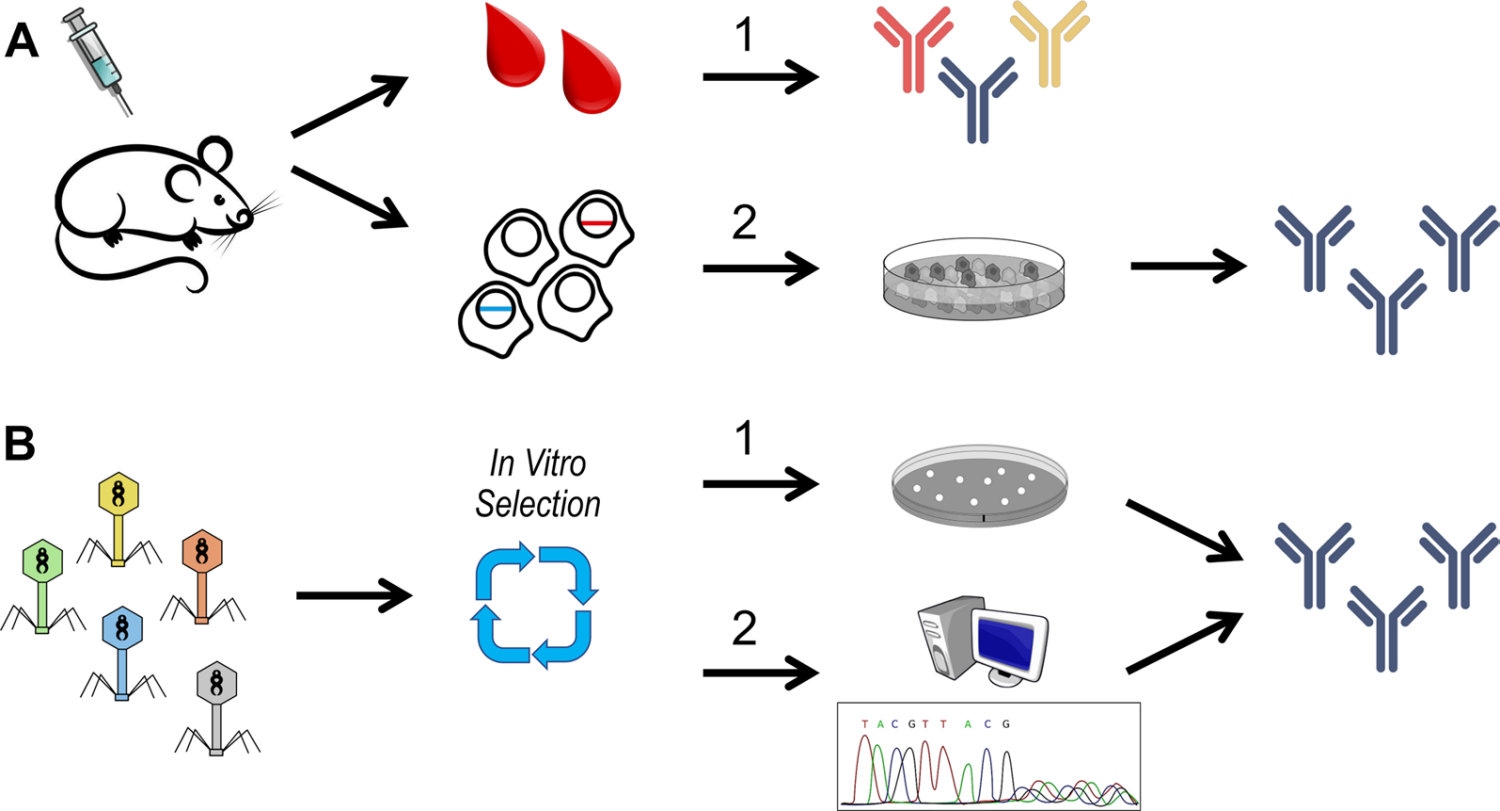
Schematic describing natural versus synthetic Ab generation strategies. The top schematic shows the generation of natural Abs via animal/human immunizations followed by either isolation of polyclonal Abs directly from blood serum (**A1**) or isolation of monoclonal Abs via hybridoma technologies involving the isolation and immortalization of monoclonal B-cells grown in culture (**A2**). The bottom schematic indicates the generation of synthetic Abs by molecular display methods (i.e. phage, yeast, ribosome, etc.). This example shows a phage-display synthetic Ab library undergoing in vitro selections against a target antigen. The selection is performed during multiple rounds to eventually enrich for antigen selective Ab binders. Individual phage clones are then isolated and via recombinant methods monoclonal synthetic Abs are generated (**B1**). More advanced methods utilize next-generation sequencing combined with advanced computational assessments to determine the clonal population profiles. Based on mathematical rankings, statistics, and various other computational approaches, individual clones are selected for recombinant protein expression, ultimately producing monoclonal synthetic Abs (**B2**)

The incorporation of deep sequencing into the design and development of synthetic Abs has significantly disrupted current biotechnological methods. For instance, deep sequencing has played a crucial role in expediting the discovery of antigen selective Abs, where it is now possible to sequence the Ab repertoire of an individual and quickly obtain the precise sequences linked with a particular immunogen/disease [20–22]. Such level of information helps guide the processes of synthetic Ab design. Furthermore, deep sequencing can play critical functions during in vitro Ab selections producing comprehensive sequence datasets for big data analysis (Fig. 1). For instance, during in vitro selection rounds, deep sequencing can be implemented to confirm the enrichment of antigen-selective clones. [23–25]. Also, the generation of NGS big data aids in assessing the efficacy of in vitro selections, helping to uncover repertoire diversity, pinpoint diversified antigen-selective clones, and elucidate the biochemical characteristics of Abs within the output selection pools. Additionally, deep sequencing procedures can be implemented to effectively determine the various biochemical patterns required for antigen interactions; such observations can be extremely valuable for the downstream engineering of synthetic Ab libraries [23–25]. Moreover, high-throughput technologies can be implemented to monitor alterations in Ab sequences across rounds of affinity maturation. Such data offers the swift evaluation of enriched clones over time, allowing for a better understanding and improvements in target binding efficacy [26].

Furthermore, deep sequencing datasets can be integrated with machine learning algorithms for synthetic Ab design. Here, machine learning refers to a broad class of computer models that have the capacity to learn from input data without being explicitly programmed. As such, machine learning-based algorithms can be introduced to exploit the enormous sequence space (composed of hundreds of millions of Ab sequences) obtained by deep sequencing experiments [27, 28]. In this context, advanced computational tools have the capacity to effectively predict antigen-selective Ab sequences; thereby expediting the processes of synthetic Ab discovery and development. When taken together, the integration of these state-of-the-art technologies including synthetic Abs, deep sequencing, and machine learning approaches is swiftly disrupting current methodologies in diverse areas of biology and biotechnology, including immunology, diagnostics, and therapeutics. Significantly, this technological integration is paving the way for precision medicine and customized treatments, helping to open new avenues for personalized healthcare [29, 30].

## Improved synthetic Ab library designs

Deep sequencing can be used as a validation tool for the design and engineering of synthetic Ab libraries [31, 32]. Its implementation offers advantageous statistical insights concerning amino acid distributions at complementary determining regions (CDRs); such quantifiable observations ultimately help determine the critical residue positions associated with antigen interactions. Additionally, it can be an effective tool to understand the actual clonal diversity, completeness, redundancy, and biases found within Ab libraries; as such, this information helps improve/optimize the final design of a synthetic Ab library final [33–35]. More recently, deep sequencing has been introduced to help validate specific sequence-encoded Ab features, this has enabled the rapid optimization of Ab sequence lengths for improved antigen recognition [36, 37]. Also, deep sequencing can provide quantitative insights from natural Ab repertoires, such information helps identify the critical features responsible for structural integrity, solubility, and affinity interactions. These observations can then be transferred to improve the design of synthetic Ab libraries [38, 39]. When taken together, big data explorations offer a quantitative approach for the generation of fine-tuned and specialized synthetic Ab library designs (Table 4).

**Table 4:** List of studies associated with improved synthetic Ab library designs based on deep sequencing big data assessments

Study	Findings	Limitations
Maruthachalam et al*.* [36]	Deep sequencing of synthetic Ab libraries helped produce diversified sub-libraries with fine-tuned CDR lengths for improved target antigen recognition	▪ Uses a single-framework synthetic Ab library ▪ The only diversified CDRs include H1–H3 and L3 ▪ Target antigen dependent
Chen et al*.* [39]	The authors generated a synthetic Ab library against enterovirus antigens. Deep sequencing analysis reveals that heavy chains of the enterovirus-specific Abs are conserved	▪ The Ab library is based on peripheral blood samples of enterovirus-infected donors
Chen et al*.* [40]	The deep sequencing of immunized mice enabled the reverse-engineering of an Ab response while helping guide the construction of a synthetic Ab library containing natural features	▪ The synthetic library complexity is limited to natural Ab repertoires
Larman et al*.* [41]	The successful development of a rationally designed synthetic Ab library was used in downstream applications involving deep sequencing-associated Ab discovery	▪ Limited to a ribosome-display format ▪ The only diversified CDRs include H2–H3 and L3
Tiller et al*.* [42] Ravn et al*.* [43] Frigotto et al*.* [44]	These studies demonstrate the effective large-scale, quality controlled validations of engineered synthetic Ab libraries by extensive deep sequencing analysis	▪ The longer reads show reduced data quality due to greater CDR complexities and read-out errors
Li et al*.* [48]	A machine learning model, trained on extensive deep sequencing datasets, was able to effectively engineer various synthetic Ab libraries in silico. These are highly diverse and target-specific	▪ Requires target binding data for supervised training ▪ Demands supervised fine-tuning of pretrained language models
Shuai et al*.* [49]	This study presents generative language model employs bidirectional context for designing Ab sequence spans of varying lengths. It led to the effective in silico design of synthetic Ab libraries containing desirable biophysical features	▪ Requires data training from natural Ab sequences for targeted infilling of residue spans and full-length sequence generation ▪ Desirable biophysical features require the generation of large-scale full-length Ab sequences for model training
Amimeur et al*.* [50]	A machine learning model, based on a generative adversarial network, effectively generates feature-controlled synthetic Ab libraries. The method included transfer learning; thus enabling chemical and biophysical biases	▪ The proof-of-concept validation library only contains 100 k sequences ▪ Validation of the library failed to include antigen selections and identify target selective Abs
Shin et al*.* [51]	An autoregressive generative machine learning model was able to predict functional Ab sequences for the engineering of synthetic nanobody libraries. These possessed high levels of Ab expression, solubility, and stability	▪ Restricted to nanobody libraries ▪ Based on a small library that requires further affinity maturations to identify strong Ab binders

The work by Chen et al*.* highlights how natural Ab features can be designed into synthetic Ab libraries for improved activities [40]. To accomplish this, the authors performed deep sequencing analysis of Ab repertoires from both naïve and immunized mice. Via this process, they were able to determine the critical Ab sequence features associated with target antigen affinity interactions. In this context, the implementation of big data analysis aided in identifying enriched amino acid frequencies within particular CDRs. These characteristics were then incorporated into the construction of a completely synthetic Ab library. Following in vitro selections using this library, the authors successfully isolated unique clones exhibiting improved affinities when compared to Abs derived from in vivo immunological responses [40]. The functionality of their synthetic Ab library was further validated by performing multiple in vitro selections against diversified antigens. Via this process, the authors identified a complete set of selective Abs against distinct molecular targets [40].

Overall, Chen et al*.*'s findings indicated that their synthetic Ab library possessed broad reactivity, extending beyond a singular antigen or molecule family. Additionally, their results demonstrated that the prevalent canonical structure combination of CDRs derived from mouse Ab repertoires serves as a robust framework for generating highly effective Abs against diverse protein antigens. Collectively, Chen et al*.*'s research illustrated the potential for designing synthetic Ab libraries based on predominant CDR characteristics observed in natural Ab repertoires. This approach, utilizing deep sequencing-based big data alongside statistical analyses, holds significant promise in synthetic Ab library construction. Particularly, in the identification of sequence features through bioinformatic methods proves essential in developing fully functional synthetic Ab libraries, catering to a wide array of target antigens. Thus, synthetic Ab libraries based on natural Ab repertoire features can yield high affinity Abs, capable of targeting various antigens across diverse epitopes.

In an independent study, Larman et al*.* focused on developing a rationally designed synthetic Ab library intended for subsequent application in deep sequencing-associated Ab discovery [41]. To accomplish this, the authors constructed an in silico*,* fully defined single-chain variable fragment (scFv) library. Also, to streamline downstream deep sequencing data analysis they introduced specialized sequence features into the scFv framework [41]. Another key feature is that they included minimal variable region diversifications, just focusing on specific CDRs (L3 and H2-H3). Additionally, they fine-tuned their unique CDR sequence design for protein binding by incorporating a hidden Markov model trained on thousands of Ab-antigen cocrystal structures obtained from the Protein Data Bank [41]. Consequently, their computational method was able to delineate the crucial CDR positions essential for antigen binding, culminating in the creation of a synthetic scFv library founded on strategically designed variations that complemented the antigen interactions observed in natural Abs.

To materialize their synthetic library, Larman et al*.* synthesized all unique in silico sequences on a programmable microarray. Subsequently, these fragments were combinatorially cloned into a unified scFv framework for molecular display [41]. With a rationally designed synthetic Ab library on hand, the authors then proceeded to conduct in vitro selections against various cancer-associated antigens. To confirm the efficacy of their library, they conducted deep sequencing analysis on all selection output pools. Due to the construction features originally designed in silico, the authors were then able to multiplex the output paired-ended reads to effectively conduct comprehensive computational Ab sequence profilings. In turn, this enabled them to pinpoint the highly-ranked clones associated with antigen interactions. Additionally, their statistical analysis also served to validate that a rationally constructed scFv library was proficient at generating a diverse range of Ab binders targeting distinct antigens [41]. When taken together, the work presented by Larman et al*.* highlights a paradigm shift in synthetic Ab library design, revealing how in silico-derived Ab libraries can be engineered for downstream applications involving big data Ab discovery.

Moreover, the utilization of deep sequencing technologies presents significant advantages for conducting quality control in the design of synthetic Ab libraries [42–44]. Here, big data explorations provide a statistically-driven framework to corroborate specific Ab-engineered features, such as rationally designed clonal diversities. Furthermore, deep sequencing can be implemented during each round of in vitro Ab selections to effectively assess the quality of the selection process. In this context, big data assists with tracking the evolution and expansion of specific clones within a population over the selection process, providing a snapshot of Ab clonal enrichments and concentrated diversities [26, 45]. An example of this feature includes the work by Ravn et al*.* Here, the authors implemented deep sequencing coupled with bioinformatic analyses to assess the clonal diversities and Ab sequence quality of their engineered synthetic Ab libraries [43]. To do this, they performed NGS following each round of in vitro selections against distinct target antigens. When computationally assessed, the various deep sequencing datasets could be used to effectively determine the evolution of Ab frequencies and clonal enrichments. Furthermore, bioinformatic explorations of big data enabled the clustering and parsing of all unique clones into specific family lineages; this led to the rapid discovery of diversified top-ranking candidate Abs associated with target antigen interactions. As such, after experimental validations, all selected high ranking Abs (derived from distinct lineages) revealed potent interactions against their antigen targets [43]. When taken together, the work by Ravn et al*.* demonstrated how big data explorations allow for enhanced quality control during both, library design and target selections. Ultimately, their approach enabled the streamlined discovery of lead candidate Abs.

More recent advances combine deep sequencing-derived datasets with advanced computational models to improve the features of synthetic Ab libraries. An example includes the work by Li et al*.* Here, the authors utilized deep sequencing Ab datasets (combined with biochemical measurements involving Ab-antigen interactions) for the supervised training of a machine learning algorithm [46, 47]. After training their model, the authors were able to effectively extrapolate a Bayesian-based Ab fitness landscape [48]. After extensive computational iterations and validations, Li et al*.* could derive various synthetic Ab libraries completely in silico. In their computational design they included potential Ab affinity-diversity trade-offs, as well as introduction of biochemical features beyond affinity, these included hydrophobicity and isoelectric point enhancements [48]. Importantly, when experimentally validated, their in silico-designed Ab libraries yielded multiple target selective Abs, with some exhibiting sub-nanomolar binding affinity clones [48], a relevant feature for therapeutic Ab development. When taken together, the work presented by Li et al*.* revealed how a machine learning model, trained on extensive deep sequencing datasets and biochemical data, was able to successfully engineer diversified synthetic Ab libraries. Moreover, when experimentally validated, the in silico-derived libraries produced unique Ab clones with favorable target binding affinities and diversities.

In a different work, Shuai et al*.* showed how it was possible to develop a deep generative language model, termed IgLM, for the in silico generation of synthetic Ab libraries [49]. Here, their work focused on the redesign of the variable-length regions in Ab sequences. Initially, their computational model was trained using 558 million deep sequencing Ab reads. To further condition their model, they incorporated information concerning specific Ab chain usage and species-of-origin [49]. Also, their generative language model included a bidirectional context, this allowed for the design of Ab sequence spans containing varying lengths. Furthermore, their deep-learning model was also designed to predict sequences based on the infill of CDR loops for improved developability [49]. As such, their computational approach led to the effective in silico design of diversified full-length heavy and light chain Ab sequences [49]. Its predictions were compiled to design in silico synthetic Ab libraries containing desirable biophysical features. Thus, Shuai et al*.* produced a deep generative language model for creating synthetic Ab libraries based on re-designing variable-length spans of Ab sequences. Importantly, their methodology for Ab design effectively included an autoregressive sequence generation task based on text-infilling in natural language. When taken together, their work stands as a proof of concept highlighting that deep sequencing datasets and advanced computational algorithms can be combined for the engineering of in silico Ab libraries, where these can be fine tuned for desired features.

In a different work, Amimeur et al*.* describe a generative adversarial network capable to design feature-controlled synthetic Ab libraries [50]. Briefly, the authors trained their computational algorithm by utilizing over 400,000 deep sequencing light- and heavy-chain human Ab sequences [47]. This training approach helped to computationally define the rules of human Ab formation and residue features. Also, their machine learning model incorporated transfer learning, a computational strategy that adds biases in the generative adversarial network towards molecules with desired features. For instance, the authors included in their design improved stability and developability, lower predicted major histocompatibility complex (MHC) Class II binding, and biases toward specific CDR residues [50]. After extensive data analysis and complex iterative computations, Amimeur et al*.* were capable to design in silico synthetic Ab libraries that contained natural Ab features. Following, they validated their computational method by successfully expressing a proof-of-concept library of nearly 100,000 Abs via phage display. After experimental validations, the in silico-designed Abs proved structurally stable and possessed high solubility index scores, while containing correct/desired molecular design features [50]. Taken together, the work presented by Amimeur et al*.* effectively demonstrates how a generative adversarial network is capable to design feature-controlled synthetic Ab libraries. Specifically, it is able to capture the complexity of the natural Ab sequence space, providing a basis for the generation of novel Abs that span a larger sequence diversity than those explored by standard in silico generative approaches. Also, through transfer learning, their model provided an inherent method to bias the physical properties of the generated Abs toward improved developability and biochemical properties.

An alternative strategy includes the work by Shin et al*.* Here, the authors showed how an autoregressive generative model can help predict novel Ab sequences for the engineering of synthetic nanobody libraries [51]. Briefly, the authors produced a fully unsupervised, alignment-free deep generative model (adapted from natural language processing) for the prediction of diverse Ab sequences. To train their model, they used big data generated from deep sequencing natural Ab repertoires. As such, by employing their autoregressive model they were then able to successfully gain insights into the different Ab constraints linked to functionality. Moreover, their machine learning approach helped generalize regions of sequence space traditionally considered beyond the reach of prediction and design. Ultimately, this information led to the effective prediction of specific residue positions in Ab sequences, especially those associated with missense and indel effects [51]. Following the analysis of extensive Ab datasets, the authors were then able to design synthetic nanobody libraries completely in silico [51]. As such, subsequent experimental validations using a proof-of-concept nanobody library revealed it possessed high levels of Ab expression, solubility, and stability. For example, their test 10^5^-nanobody library showed better expression levels than a 1000-fold larger synthetic library [51]. Altogether, the work presented by Shin et al*.* revealed an autoregressive machine learning model capable to generalize regions of Ab sequence space traditionally considered beyond the reach of prediction and design. Importantly, their predictive approach was able to produce functional synthetic nanobody libraries completely in silico.

## Heightened characterization of Abs

Deep sequencing is an effective tool for the characterization of synthetic Abs at high resolution. It enables the comprehensive assessment of Ab sequences, encompassing diversity, binding properties, and functional features [23–25]. Here, the combination of big data with advanced computational models offers a statistical framework to better characterize Abs (Table 5). Moreover, because deep sequencing offers the comprehensive exploration of Ab sequence space, it is able to unveil the distinct clonal features and diversities to help identify potential candidate Abs. For instance, it offers a quantitative strategy to parse and select the top ranking immuno-dominant sequences in silico, bypassing labor intensive screenings and validations [52]. A further advantage is that deep sequencing-based Ab discovery can effectively identify both unique and rare clonal sequences associated with Ab-antigen interactions [53, 54]. In this context, this approach broadens the pool of candidate Abs available, enabling the comprehensive scrutiny of clones.

**Table 5:** List of studies associated with heightened characterization of Abs based on deep sequencing big data assessments

Study	Findings	Limitations
Yang et al*.* [52] Gallo et al*.* [53] Barreto et al*.* [54]	The deep sequencing of in vitro Ab selections enabled the rapid discovery of highly diversified and rare positive clones	▪ The quality of computational analysis is dependent on target antigen, sample preparation, and background noise removal
Kelil et al*.* [55]	The introduction of a motif-based scoring algorithm was used for analyzing deep sequencing datasets derived from in situ Ab selections against cell surface receptors. It enabled effective mining of low-frequency Ab clones (i.e. rare clones) hidden in big data	▪ The quality of computational analysis is dependent on target antigen, sample preparation, and background noise removal ▪ Sufficient amount of data is required for performing motif-based statistical computations
Tsioris et al*.* [57]	A humoral response in infected subjects with West Nile virus is monitored using single-cell BCR deep sequencing analysis. This approach led to the rapid identification of viral-specific Ab clones	▪ Focuses on natural Abs ▪ Requires infected subjects to derive antigen-selective Abs ▪ Time and resource intensive
Pan et al. [62]	Isolated bone marrow from immunized mice was subjected to deep sequencing analysis. The introduction of advanced computational screenings helped identify antigen-specific clonal lineages, including Ab frequencies, where these helped determine lead candidate Abs	▪ Focuses on natural Abs ▪ Requires infected subjects to derive antigen-selective Abs ▪ Time and resource intensive
Forsyth et al*.* [66]	A deep sequencing-based CDR mutagenesis scanning platform was used to comprehensively understand clonal sequence affinities. It allowed for the rational design of synthetic Ab sub-libraries and for performing mutagenesis improvements of specific Ab variants	▪ The point mutations are dependent on experimentally pre-determined structural and mutational data ▪ It is low-throughput and based on time intensive in vitro selections, due to Ab library being displayed on mammalian cells and sorted via flow cytometry
Friedensohn et al*.* [27]	A deep-learning model trained on deep sequencing Ab data aided in the effective discovery of convergent features from distinct immune responses. The convergence of Ab features could be determined even if analyzed from distinct immunizations and antigens conditions	▪ The deep sequencing training datasets are restricted to Ab repertoires of immunized animals ▪ Convergence analysis is only focused on CDRs H1-H3 ▪ It is low-throughput and based on time intensive in vitro selections, due to Ab library being displayed on mammalian cells and sorted flow cytometry

In recent years, developments in deep sequencing-based Ab discovery have utilized advanced bioinformatic processes to effectively mine low-frequency Ab clones (i.e. rare clones) hidden in big data. As an example, the recent work presented by Kelil et al*.* describes an advanced motif-based scoring algorithm used for analyzing deep sequencing datasets [55]. In their work, the authors effectively showed how in silico methodologies are able to comprehensively interrogate the clonal output from in situ Ab selections against cell surface receptors [55, 56]. Briefly, the authors performed multiple rounds of in situ cell-based selections to enrich target selective Ab-phage pools. Following, they implemented deep sequencing analysis from Ab pools derived from both, antigen expressing cells and controls. To effectively deal with the background noise inherent to the highly heterogeneous, in situ-derived Ab-phage pools, the authors developed a motif-based scoring algorithm that explored all possible sequence paratope motifs (i.e., linear information). Here, for each Ab clone they explored the entire space of linear information by exhaustively enumerating all possible motifs matching their CDR sequences. Ultimately, Kelil et al*.* were able to obtain the frequencies (number of matching sequences/total number of sequences) of every motif in the antigen selective versus control datasets [55].

In turn, their computational methodology enabled Kelil et al*.* to compare and contrast the levels of enrichment motifs derived from Abs in the in situ selections versus controls. Such strategy successfully identified candidate Ab sequences potentially reactive for the target antigen. Importantly, their motif-based scoring model could effectively determine highly diverse Abs beyond the limitations of frequency. This meant that the various in silico identified clones had extremely diversified frequencies, these varied from very high (30%) enrichment to as low as 1 per 1,000,000 sequence reads [55]. Crucially, when experimentally validated, all of the identified low frequency Abs (i.e. rare clones) showed high selectivity for their cognate antigen. This implied that buried within deep sequencing Ab datasets lie highly diverse low-frequency clones that are specific for their antigen. Another feature from their model is that all identified rare clones had unique sequences, all divergent from the immunodominant classes [55]. This highlights that their methodology effectively expands the available pool of antigen-selective Abs. When taken together, the work by Kelil et al*.* described an advanced in silico strategy able to screen complex pools of in situ selecion Abs via deep sequencing big data mining. Here, the implementation of an advanced motif-based scoring algorithm was able to effectively mine and resolve low frequency clones selective for their target antigens.

Additionally, deep sequencing can also be used as a tool to effectively analyze the sequence diversity and evolution of synthetic Abs, such as those involved in the neutralization of pathogens. Notably, implementing this strategy allows for the swift identification of lead candidate Abs for downstream therapeutic assessments. The effective implementation of this approach was demonstrated by Tsioris et al*.*; here, the authors monitored a humoral response over time in infected subjects with West Nile virus using single-cell B-cell receptor (BCR) deep sequencing analysis [57]. Via the implementation of computational assessments, the authors could track over time the Ab immune response associated with the West Nile virus. Importantly, by observing clonal diversities and increased frequencies their findings also led to the effective identification of antigen-specific Ab clones. Furthermore, the implementation of computational ontogeny assessments helped identify the antigen-selective clones with the highest viral neutralization activities [57]. Similarly, to rapidly identify function-specific Abs, the work presented by Zhu et al*.* introduced a deep sequencing analysis to B-cell transcripts derived from patients infected with the human immunodeficiency virus-1 (HIV-1) [58]. To effectively accomplish this, they derived a computational algorithm that analyzed specific sequence motifs and Ab evolutionary relatedness. Here, via metadata analysis of deep sequencing datasets, the authors were able to swiftly identify selective candidate Abs with high sequence diversities [58]. Also, based on their findings, they were able to derive chimeric synthetic Abs using only the in silico identified heavy chain Ab sequences; these were then combined with the light chain of a previously isolated anti-HIV-1 Ab. When experimentally validated, all the reconstituted Abs showed high neutralization potencies against HIV-1, with some clones able to neutralize viral infections up to 90% [58].

Besides being utilized as a tool to explore the breadth of the selection output and clonal sequence diversities, deep sequencing datasets can be used for predicting Ab binding affinities [59–61]. Such feat is commonly done by analyzing deep sequencing datasets for clonal enrichments derived from in vivo immunizations or in vitro Ab selections. Because clonal abundances tend to correlate with affinity, the incorporation of clonal frequency rankings offers an indirect strategy for predicting Ab affinities. For instance, this was successfully demonstrated by Pan et al. where the authors isolated bone marrow from immunized mice followed by Ab repertoire deep sequencing analysis [62]. Post computational screenings, they were able to effectively identify the distinct molecular features from antigen-specific clonal lineages. Moreover, they also implemented calculations associated with enrichment rankings of the various Ab frequencies to effectively identify lead candidate Abs. Notably, when experimentally validated, all top-ranking identified clones proved to be selective for the target antigen and displayed high affinities [62]. Thus, the work presented by Pan et al. successfully demonstrated that the application of deep sequencing-based clonal rankings can effectively formulate affinity predictions; this is accomplished by performing frequency cross-comparisons among the various clones.

Additionally, deep sequencing technologies can help monitor the mutagenesis status of CDRs to ultimately enhance Ab-antigen affinity interactions. For instance, by correlating Ab sequences with affinity data or other effector properties, desired Ab variants can be swiftly identified and selected for downstream in vitro validations [63–65]. Furthermore, the implementation of computational comparative sequence analyses can help identify specific amino acids associated with improved antigen interactions, these can include critical structural locations and/or charge properties on the Ab framework [63–65]. Thus, big data comparative analyses can facilitate the discovery of sequence motifs with advantageous biophysical properties. As an example, the work presented by Forsyth et al*.* revealed how deep sequencing analysis could help characterize the Ab affinity regions involving CDR mutagenesis studies [66]. Here, their method uses Ab libraries containing thousands of CDR point mutations displayed on mammalian cells; these were then sorted by flow cytometry into subpopulations based on antigen affinity and subsequently analyzed via deep sequencing.

Next, by performing clonal enrichment rankings, the authors were able to determine the various mutations associated with affinity improvements. Their analytical approach assessed the effect of every possible amino acid CDR substitution for potential antigen binding interactions. Ultimately, streamlining the identification of Abs with enhanced affinity [66]. As a proof-of-concept, they applied their method to a humanized Ab version of the anti-epidermal growth factor receptor, called cetuximab. Then they generated a comprehensive dataset that included 1060-point mutations that recapitulated previously identified structural and mutational data for these CDRs. In turn, the implementation of their computational and experimental findings helped reveal 67 critical point mutations associated to increase the affinity of cetuximab [66]. When taken together, the work presented by Forsyth et al*.* highlights a deep sequencing-based CDR mutagenesis scanning platform for the comprehensive understanding of clonal affinities. Moreover, their work may prove extremely advantageous for the rational design of Ab sub-libraries and for performing mutagenesis improvements of specific Ab variants.

Additionally, the combination of deep sequencing datasets with advanced computational algorithms can aid in the characterization of synthetic Abs. As an example, Friedensohn et al*.* showed how a deep-learning platform capable to identify convergent features among distinct Ab repertoire pools [27]. Notably, their work provided evidence for the presence of shared (convergent) receptor sequences across organisms of the same species. Briefly, the authors introduced a deep-learning method, based on variational autoencoders, to help model the underlying processes of BCR recombinations. For training their algorithm they used deep sequencing-derived datasets from Ab repertoires involving immunized mice cohorts [27]. Following, their advanced computational algorithm performed latent embedding and the clustering of assigned labels to help group similar sequences. Such analysis allowed for the discovery of convergent, antigen-associated sequence patterns. Following, they confirmed their findings by performing in vitro validations involving both natural and in silico-generated Abs with convergent patterns. Here, their experimental results showed that all novel in silico-derived Abs exhibited high binding affinities for their target antigens [27]. Thus, the work by Friedensohn et al*.* effectively highlights how a machine learning model, trained on deep sequencing datasets, can aid in the discovery of convergent features from distinct immune response Ab repertoires. Importantly, their findings show that this occurs even if those convergent features are based on distinct ranges of immunizations and antigen conditions [27].

## Enhanced affinity maturation strategies

Deep sequencing offers critical information for the design of affinity maturation libraries to successfully improve Ab-antigen interactions. For instance, via deep sequencing it is now possible to guide and monitor the changes in Ab repertoires over rounds of affinity maturation; such feature helps track the entire clonal output and determine affinity improvements over time [26]. In this context, it can offer a statistical overview of the unique clonal repertoires. This capability is pertinent for conducting clonal frequency assessments to ascertain the efficacy of in vitro selections, including the presence of immuno-dominant high-affinity Abs [59–62]. Furthermore, this real-time feedback offers opportunity for instant responses regarding modifications in the Ab library design [67]; in turn, this can help avoid potentially cost and time-consuming molecular evaluations. Also, deep sequencing explorations can help determine the representation of distinct Ab library features associated with antigen interactions. These observations can then be analyzed via computational models to determine Ab-antigen interactions and molecular docking predictions, ultimately enabling a holistic approach to big data assessments [68, 69]. Moreover, such in silico predictive models can offer valuable insights to improve the designs of affinity maturation libraries.

The work by Hu et al*.* successfully showed how a deep sequencing big data platform could be used to effectively identify Ab candidates with improved affinities [60]. Briefly, their methodology involves the deep sequencing of the clonal output from affinity maturation libraries. These were then computationally analyzed and clustered based on clonal rankings of the various frequencies. Next, the authors selected the top candidate clones based on their enrichment and uniqueness. As a consequence, their approach was able to effectively streamline the affinity maturation process, where it allowed to bypass the requirements for primary screenings. Also, they corroborated their findings by designing a combinatorial sub-library that included specific biochemical patterns and amino acid positional frequencies at each CDR. These were derived from the unique features contained in the ten most abundant and diversified Ab variants. Importantly, after in vitro selections against a target antigen, they were able to rapidly identify and isolate a mutant clone with 158-fold improved affinity from the parental clones [60]. When taken together, the work presented by Hu et al*.* helped demonstrate how deep sequencing datasets can aid in the swift discovery of affinity matured clones. Moreover, the application of deep sequencing could effectively discern the specific CDR features associated with target antigen interactions, allowing for the construction of combinatorial sub-libraries that generate clones with significantly improved Ab affinities.

Furthermore, deep sequencing can help identify specific amino acid positions or motif-regions associated with Ab-antigen interactions. For instance, the work by Fujino et al*.* revealed a robust deep sequencing-based engineering strategy that helped derive synthetic Abs with enhanced affinities [70]. Briefly, the authors first generated a single amino acid mutational scanning Ab library (based on a previously identified Ab) to identify the affinity binding hotspots. This scanning library was then used for performing in vitro selections against the target antigen, with the output repertoire analyzed by deep sequencing. After rigorous computational analysis, Fujino et al*.* were able to determine the critical residues associated with antigen binding [70]. Based on the identified CDR hotspots, the authors further engineered a combinatorial sub-library for performing downstream affinity maturations. After in vitro selections using the mutagenesis-guided sub-library, a small subset of enriched clones was quickly isolated, where post experimental validations all distinct clones exhibited strong interactions with the target antigen. Notably, some Abs exhibited affinities greater than 2000-fold from the parental clones [70]. When taken together, Fujino et al*.* described an innovative deep sequencing big data strategy for enhancing the affinity of Abs in vitro. Their methodology produced several pico-molar affinity clones, an important feature for the development of Ab therapeutics.

Moreover, the introduction of advanced computational models can be leveraged to successfully enhance Ab affinities. As an example, the work by Saka et al*.* revealed how a completely in silico Ab discovery platform is able to derive novel high-affinity Abs [71]. Briefly, the authors derived an advanced machine learning method capable to decipher the distinct CDR patterns associated with high-affinity antigen binding interactions. To train their computational algorithm, the authors incorporated expansive deep sequencing Ab datasets from distinct in vitro experimentations. After big data processing, their machine learning model could effectively predict novel synthetic Ab sequences that were associated with high-affinity interactions. This was done by introducing a Long Short Term Memory (LSTM) network capable to generate and prioritize Ab sequences with high affinity interactions. This deep generative model uses natural language processing, where after training, it can sample virtual sequences and avoid combinatorial features commonly encountered in the deep sequencing space. As such, the authors prioritized and selected the most promising virtual sequences based on their calculated likelihoods. Eventually, all high-ranking in silico-derived clones underwent experimental validations; importantly, all predicted Abs displayed potent affinities for their target antigens [71]. A distinctive feature of their approach was that most of the in silico predicted clones had exceptionally improved affinities (some 1800-fold higher) when compared to the identified clones derived from the original deep sequencing data [71]. When taken together, the work by Saka et al*.* showed how an advanced machine learning algorythm can be implemented to generate completely novel Ab sequences that are target selective and high affinity.

A current downside of conventionalAb affinity maturation strategies is that these prove overly costly and labor-intensive. This is due to the molecular mutational rearrangements required to effectively explore the sequence space associated with improved affinity interactions. To overcome this current limitation, Liu et al*.* developed a deep generative model, based on Ab sequence generation and prioritization procedures, to efficiently predict Ab sequences with higher affinity [72]. Briefly, the authors first performed in vitro selections against a target antigen using a synthetic Ab library with varying CDR3 sequences. At each selection round, they generated extensive deep sequencing datasets that were used for training their deep-learning algorithm. Following, they introduced LSTM network calculations capable to make effective in silico predictions involving Ab-antigen affinity interactions. Notably, their de novo predicted clones proved superior in target affinity binding when compared to all of the sequences present in their deep sequencing training data [72]. For instance, some of the affinities measured displayed 1800-fold higher potencies than those of the parental clones [72]. In turn, the work by Liu et al*.* revealed how a high-capacity deep-learning algorithm can efficiently model the biophysics of Ab-target interactions given sufficient high-quality training data. Moreover, when compared to frequency-based screenings using deep sequencing datasets, their machine learning approach is able to predict de novo sequences with far greater affinities.

In summary, big data plays a pivotal role in developing affinity maturation strategies (Table 6). For instance, deep sequencing provides a window for the detailed understanding of sequence diversities, structure–function relationships, and the evolutionary dynamics of distinct Ab clones. Also, big data explorations aid in the engineering of affinity maturation sub-libraries; in turn, these help refine the specific designs and characteristics embedded in the sub-library repertoires, ultimately generating clones with improved affinities. Moreover, the introduction of deep sequencing into affinity maturation Ab discovery can help expedite the identification of high affinity Abs (in addition to other desired biochemical features). Also, deep sequencing big data is able to effectively train advanced computational algorithms to eventually produce in silico-predicted Ab affinity clones. Such de novo variants not only contain novel sequence features, but possess improved binding interactions with their target antigens. Thus, when employed together, big data and advanced computational tools can help streamline lead candidate Ab development (including affinity maturation); this is especially advantageous for biotechnology Ab production pipelines.

**Table 6:** List of studies showing enhanced affinity maturation strategies based on deep sequencing big data assessments

Study	Findings	Limitations
Hu et al*.* [60]	The deep sequencing of affinity maturation Ab libraries allowed for an in-depth evaluation of the enrichment landscapes in CDR sequences and amino acid substitutions. Here, potent affinity candidates were identified according to their high frequencies, by-passing the need for experimental primary screenings	▪ Uses mutagenesis libraries of single CDRs (either L1, L3, and H1–H3) ▪ For generating high affinity clones it requires the development of combinatorial sub-libraries to explore the synergistic effects of the different CDRs
Reddy et al*.* [61]	Deep sequencing and bioinformatic analyses were used to mine Ab variable region repertoires from plasma cells of immunized mice. Following, the pairing of the most abundant variable heavy (VH) and variable light (VL) genes, based on their relative frequencies, produced nanomolar affinity Abs	▪ Based on natural Ab repertoires derived from animal immunizations ▪ Requires further Ab reconstructions, based on pairings of VH and VL genes and subsequent in vitro screenings
Pan et al*.* [62]	Mature B cell repertoires from immunized mice were used to generate yeast display Ab libraries. After in vitro selections followed by deep sequencing analysis the authors were able to elucidate the affinity and molecular features of antigen-specific clonal lineages	▪ Based on natural Abs from bone marrow and spleen ▪ Uses Ab transgenic immunized mice ▪ Ab lineages based only on CDR-H3 clustering
Fujino et al*.* [70]	A mutational scanning Ab library was subjected to in vitro selections followed by deep sequencing analysis. This allowed the identification of all the critical residues associated with antigen binding. Subsequently, based on the identified CDR hotspots, a combinatorial sub-library was constructed to generate high affinity clones	▪ Uses the Ab sequence of a previously identified Ab ▪ Mutagenesis only focuses on 50 amino acid positions comprising all CDR loops ▪ Requires the generation of sub-libraries to identify high affinity Abs
Saka et al*.* [71]	A machine learning model was capable to decipher the distinct CDR patterns associated with high-affinity antigen binding interactions in deep sequencing datasets. This model was able to sample virtual sequences and avoid combinatorial features commonly encountered in the deep sequencing space. The in silico predicted clones had unique sequences with exceptionally improved affinities	▪ Uses a synthetic heavy chain library with limited sequence diversity ▪ Requires Ab-antigen in vitro experimentations ▪ The deep sequencing training data contained non-specific binder sequences, and true binder sequences were never determined
Liu et al*.* [72]	A deep generative model, based on Ab sequence generation and prioritization, was trained on deep sequencing big data to effectively generate Ab sequences with higher affinity interactions. Its predictions can be generalized to produce de novo Ab sequences with improved affinities	▪ Clone predictions were based on computing the minimal sets of specific CDR-H3 amino acids required for binding ▪ Requires primary Ab campaigns and subsequent affinity maturation steps to achieve desired affinities

## Reduced Ab immunogenicity effects

The generation of deep sequencing datasets from either immunized animals or in vitro selections can effectively streamline the Ab discovery process. However, a problem arises when non-human origin Abs are intended for use in human hosts; there is a risk for inducing an immune response, causing the rapid clearance of the non-human origin Abs. To avoid such issues, non-human Abs tend to undergo a process called “humanization”. Via this approach, the non-human molecular Ab features are re-configured to resemble human ones minimizing their immunogenicity potential [73, 74]. A common Ab humanization technique involves CDR grafting, a procedure that involves the swapping of human Ab CDRs for non-human CDR counterparts [75, 76]. Although this approach has been proven mostly effective, it can be suboptimal and has important drawbacks. For instance, the resulting CDR-grafted chimeric Abs may require further molecular engineering and mutagenesis optimizations. This is done to ensure that their desired biochemical properties remain undisturbed, such as affinity, solubility, and stability. Also, the retention of non-human CDR regions in a human Ab framework can potentially induce immunogenicity in some human hosts. Thus, due to CDR-grafting into human framework regions of germline sequences drawbacks, recent developments have employed big data coupled with sophisticated computational techniques to effectively perform in silico Ab humanizations.

For instance, the recent work by Clavero-Álvarez et al*.* described a computational method, based on a multivariate Gaussian model, capable to characterize the statistical distribution of the sequences of the variable regions of human Abs [77]. Here, the authors used Ab sequence information to effectively calculate the phenotypical correlations between pairs of residues, both within and between chains. Furthermore, they implemented a probability score to assess the model’s efficiency in classifying murine and human sequences. To accomplish this they introduced large human and murine databases, obtained from the IMGT/LIGM-DB server (based on cDNA variable heavy and light Ab sequences), to help train and test their in silico model [77]. Their approach was then combined with steepest-descent/Monte-Carlo simulations to eventually generate highly accurate multivariate Gaussian statistical scores. Ultimately, their results showed that their model was able to derive humanness scores with high precision; as such, this feature enabled to perform the humanization of various murine sequences. Importantly, when validated their model outperformed other computational methods involved in sequence classifications [77]. Moreover, their optimization protocol was able to effectively generate humanized sequences that were recognized as human-like by various homology modelling tools [77]. When taken together, Clavero-Álvarez et al*.* described an advanced computational model that is capable to infer humanness features based on large datasets of experimentally verified Ab sequences. Notably, their approach offers a flexible framework that can be adapted to different learning databases to ultimately extrapolate the humanization of animal-derived Abs.

Another example includes the recent work by Schmitz et al*.* Here, the authors effectively describe a computational methodology, termed IgReconstruct, that is able to characterize the immunogenicity potential from single nucleotide frequencies contained in the Ab variable regions [78]. To accomplish this, they derived a computational model that utilizes a position- and gene-specific scoring matrix capable to generate human similarity scores. Here, their scoring matrix uses sequence back-translation comparisons, allowing for accurate similarity estimations between human and non-human Ab sequences [78]. To derive their calculations they utilized big data training sets derived from the deep sequencing of human BCR repertoires; these included approximately 326 million Ab sequences [78]. After performing computational reiterative alignments to species-specific germline genes they were able to map the various non-human Ab sequences onto the immunome of human BCR repertoires. Ultimately, their methodology helped discern the immunogenicity potential from single nucleotide frequencies of non-human Ab variable regions [78, 79]. Altogether, the work presented by Schmitz et al*.* showed how an advanced computational model, dependent on expansive deep sequencing Ab datasets, can effectively identify the key immunogenical features from non-human Ab sequences.

Similarly, the recent work by Prihoda et al*.* described how deep sequencing Ab datasets may be combined with deep-learning models to perform accurate Ab humanizations [80]. Briefly, the authors incorporated public repositories of big data Ab sequences [47] to train their in silico humanization model, termed BioPhi. Their strategy utilized a deep-learning methodology able to predict the most probable human residues given a particular input sequence. For performing accurate determination, their computational algorithm first partitioned a given Ab sequence into overlapping 9-mer peptides. Then, by introducing exhaustive comparative iterations against NGS datasets of human-derived Ab sequences the authors could determine the prevalence of human-like features from a given input sequence. Ultimately, their deep-learning algorithm was able to predict positional residue frequencies, allowing to perform guided adjustments to an input sequence. In turn, this procedure could then determine the positional Ab residue mutations associated with immunogenicity. Furthermore, after performing experimental validations, the authors showed that the in silico-predicted sequences had comparable affinities as to those generated by standard methods [80]. When taken together, Prihoda et al*.* presented a deep-learning architecture, trained on extensive deep sequencing datasets, that evaluates the “humanness” composition of a given Ab sequence. Via such method, the authors were able to perform rapid molecular engineering optimizations that led to effective Ab humanizations.

Other humanization approaches include the work presented by Wollacott et al*.*; here, the authors developed a deep-learning model, termed AbLSTM, based on extensive training using NGS-derived BCR repertoires. In their work, the authors were able to effectively identify the divergent Ab features from non-human Abs to natural human counterparts [81]. Briefly, Wollacott et al*.* developed a bi-directional LSTM network trained on extensive NGS-derived Ab sequence datasets. To advance their computational calculations they introduced a specialized recurrent neural network to effectively learn the distinct amino acid distributions found in Ab sequences. This helped determine selective pattern identifications involving long durations of time. When computationally validated, their in silico approach could effectively perform sequence classifications, allowing for the differentiation of human Abs from those of other species. As such, the immunogenicity information obtained via their computational approach helped guide the in vitro molecular humanizations of non-human Abs. Another advantage of their method is that it showed direct implications for the 'humanness' evaluation of synthesized Ab libraries, as well as at predicting the most favorable architectures for downstream CDR grafting into human frameworks [81]. When taken together, the work presented by Wollacott et al*.* described an advanced computational model, based on a LSTM network, that is capable of learning the specific native features within human Ab sequences. To assess the humanness potential of a given Ab sequence, their approach employed extensive deep sequencing datasets from naturally occurring Ab repertoires; these were used as the training vehicle to understand the characteristics associated with human Abs.

Similarly, the work by Marks et al*.* described a deep sequencing-dependent machine learning algorithm capable of discriminating human versus non-human Ab sequences [82]. Briefly, to train their model the authors used deep sequencing Ab datasets derived from over 65 million non-redundant human Ab sequences and 13 million non-human sequences [82]. Following, the authors implemented random forest classifiers to derive a quantitative scoring matrix capable of determining the human-like features given a random Ab sequence. Next, Marks et al*.* generated matrix scores based on negative comparative relationships, including experimentally validated immunogenicity effects. As a consequence, the higher the ‘humanness’ score the closer the resemblance of a given Ab sequence to a human counterpart. As such, the matrix scores helped provide a quantitative framework to determine non-human features. To effectively validate their model they incorporated random Ab sequences from previously identified therapeutic Abs. Importantly, experimental validations showed high accuracy of non-human versus human classifications; for instance, 175 of the 176 human Abs were classified as human, and all 14 mouse Abs were classified as non-human [82]. When taken together, the machine learning model presented by Marks et al*.* proved to be an effective tool for making successful humanness predictions given random Ab sequences.

Additionally, the random forest classifiers derived by Marks et al*.* were further used to help build Hu-mAb, a computational predictive tool that can systematically humanize Abs by suggesting key mutational alterations that can increase their humanness scores [82]. Notably, a key feature of Hu-mAb is that it humanizes Ab sequences by minimizing their number of mutations; this helps prevent potential unwanted sequence modifications associated with Ab function and efficacy. Thus, to humanize a given Ab sequence, their computational algorithm first assesses every possible non-destabilizing single-site mutation in the Ab framework. This then generates a large set of mutated sequences, where these are subsequently scored by random forest classifiers to derive their final ‘humanness’ scores. Following, the in silico-generated mutant sequences are ranked, with the top-scoring sequences then selected as the most human-like. Also, these mutagenized sequences all contain the minimal number of possible mutations in combination with high humanness scores. When compared to humanized Abs derived by conventional methods, the predictive tool called Hu-mAb was able to generate non-immunogenic de novo sequences just as effectively [82]. Furthermore, the mutations suggested by Hu-mAb showed substantial overlap with those deduced experimentally. This observation highlights their computational model as an effective replacement for trial-and-error humanization experiments by producing similar results in a fraction of the time. When taken together, the work presented by Marks et al*.* revealed a powerful and fully automated in silico approach capable to effectively humanize a given input Ab sequence. Furthermore, their model also helps minimize the number of mutational alteration to avoid impact on Ab efficacy, stability, and potency.

In summary, the combination of deep sequencing datasets coupled with in silico methods has radically altered the current methodologies associated with Ab humanization, processes that are time and resource intensive. On the other hand, big data Ab sequences can be used to train advanced computational models, allowing to successfully mine datasets and derive heuristic potentials. Notably, these intricate algorithms have demonstrated the ability to distinguish subtle Ab sequence differences between human and non-human Abs at an exceptional level [83–87]. For instance, various computational models have been successful at identifying position-dependent probabilities of the critical amino acid residues associated with immunogenicity hotspots [83–87]. Such approach has allowed to effectively survey non-human Ab sequences against big data phenotypic pools of human Abs; in turn, providing high throughput statistical rankings associated with reduced immunogenicity. Furthermore, big data-driven in silico Ab modeling methods can also be implemented to effectively predict de novo Ab variants containing properties of low immunogenicity [83–87], where these big data-assisted computational methodologies can transform non-human Ab sequences to closely resemble human counterparts. This means that current Ab humanization strategies can be performed completely in silico (Table 7), bypassing the need for conventional Ab humanization strategies (such as CDR-grafting), procedures that are time and resource intensive, and lack full efficacy.

**Table 7:** List of various studies highlighting various strategies for reduced Ab immunogenicity based on big data assessments

Study	Findings	Limitations
Clavero-Álvarez et al*.* [77]	The authors presented a computational method based on a multivariate Gaussian analysis that is able to characterize the statistical distribution of the variable sequences from human Abs. Their analysis was performed using large human and murine learning databases, which led to the humanization of various murine sequences	▪ The strategy is dependent on size and quality of the learning databases ▪ Only developed for humanizing murine Abs ▪ Uses stringent threshold scores that reduce the number of potential humanized sequences
Schmitz et al*.* [78]	A large immunome dataset of 326 million human Ab sequences was used to create a position- and gene-specific scoring matrix. This strategy was used to effectively analyze the human Ab sequence space, allowing for a given input sequence to be compared against associated human Ab features	▪ The scoring is exclusively calculated from V and J gene templated regions ▪ The untemplated CDR-H3 region is not included in the score calculation ▪ The scoring success depends on the chain type and CDR-H3 of certain lengths
Prihoda et al*.* [80]	A deep-learning methodology was able to predict the most probable human residues given a particular input sequence. This was done by performing exhaustive comparative iterations using NGS datasets of human-derived Ab sequences; this helped determine the prevalence of human-like features from a given input sequence	▪ The model is trained to recognize masked or mutated residues, and repairing them is based on their sequence context ▪ To compare across humanization methods, only average performance results across multiple sequences were used
Wollacott et al*.* [81]	A LSTM network was capable of learning the specific native features within Ab sequences. To effectively assess the humanness potential of a given Ab sequence, the approach was trained using extensive deep sequencing datasets from naturally occurring Ab repertoires. Ultimately, the model was successful at humanness predictions given random Ab sequences	▪ The model performance is related to the underlying sequence space used in training ▪ The LSTM model favors sequences that are more germline-like ▪ The LSTM model attributes rare sequences as being outliers
Marks et al*.* [82]	A predictive model uses machine learning classifiers that are trained using deep sequencing big data. It is then used to derive specific mutations given an input sequence to help reduce its immunogenicity potential. The predicted mutations show substantial overlap with those deduced experimentally, proving the methodology as an effective replacement for trial-and-error humanization experiments	▪ The efficiency of predictions reduces when classifying sequences of species it has not been trained on ▪ The model is intended for use on murine precursor sequences ▪ The model is not applicable for the humanization of alternative Ab formats (*e.g.* nanobodies and asymmetric Abs)

## Conclusion

The future trajectory of synthetic Ab development lies at the convergence of big data resources, involving vast Ab sequence databases and deep sequencing datasets, and cutting-edge computational algorithms adept at processing big data. This amalgamation of technologies, as explored in the present report, marks a paradigm shift in conventional methodologies ultilized for synthetic Ab discovery, engineering, and optimization. As such, by harnessing the power of these integrated tools, there is potential for broadening the scope of methodologies while reducing the requirement for resource-intensive and time-consuming procedures, both intrinsic to synthetic Ab discovery and design. Furthermore, the strategic deployment of these innovative tools holds promise to fastrack the various development pipelines employed in biotechnology and therapeutic Ab production, where researchers could potentially expedite the identification of promising candidates, hastening their progression through preclinical and clinical evaluation stages. In essence, the integration of big data resources and computational algorithms not only advances synthetic Ab development, but also offers a tangible pathway towards accelerating the delivery of therapeutic Abs.

At the same time, it is critical to emphasize that while many of the methodologies presented in this report hold high promise, these are still in their early stages of development. As such, many of these approaches demand further refinements and optimizations to eventually attain standardization within research settings. Also, it's vital to recognize that existing computational models rely heavily on extensive training and testing datasets, with their accuracy directly correlated to the quality and quantity of the available data. Thus, this dependence poses inherent limitations on their practical utility. Furthermore, the implementation of big data-driven computational analyses comes with certain considerations, particularly those concerning the time-intensive nature of data processing. For instance, numerous approaches involving extensive data processing require iterative procedures associated with significant processing times, including access to high-powered hardware resources. Consequently, the implementation of advanced computational methodologies demands careful deliberation, specifically regarding the temporal aspects of data processing and the costs associated with acquiring advanced computational hardware.

In addition to addressing these challenges, ongoing scientific efforts should also focus on enhancing the scalability, efficiency, and accessibility of computational methodologies for synthetic Ab research. Here, collaborative initiatives aimed at standardizing protocols, improving data sharing practices, and advancing computational infrastructure will be pivotal in accelerating the translation of in silico approaches into robust tools for Ab engineering. Despite these inherent drawbacks, it is evident that the integration of advanced computational models and big data analytics are effectively reshaping the landscape of synthetic Ab discovery, engineering, and development. These in silico approaches offer high potential to effectively expedite and streamline existing Ab design processes by effectively mining, filtering, and analyzing big data, including the intricate features concealed within deep sequencing datasets. As such, with the continuous evolution of computational resources and capabilities, as exemplified by Moore's law, and the exponential growth of big data repositories and databases, we should expect a disruptive quantum leap in synthetic Ab discovery, design, and development in the foreseeable future.

## Data Availability

Not applicable.

## Abbreviations

Abs: Antibodies
mAbs: Monoclonal Abs
NGS: Next-generation sequencing
CDRs: Complementary determining regions
scFv: Single-chain variable fragment
MHC: Major histocompatibility complex
HIV-1: Human immunodeficiency virus-1
BCR: B-cell receptor
LSTM: Long short term memory
